# Aversive Foraging Conditions Modulate Downstream Social Food Sharing

**DOI:** 10.1038/s41598-018-35910-6

**Published:** 2018-12-10

**Authors:** Abby Basya Finkelstein, Gro V. Amdam

**Affiliations:** 1 School of Life Sciences, Arizona State University, Tempe, USA; 2 Psychological and Brain Sciences, Boston University, Massachusetts, USA; 3 Faculty of Environmental Sciences and Natural Resource Management, Norwegian University of Life Sciences, Aas, Norway

## Abstract

Eusocial insects divide their labour so that individuals working inside the nest are affected by external conditions through a cascade of social interactions. Honey bees (*Apis mellifera)* transfer food and information via mouth-to-mouth social feeding, ie trophallaxis, a process known to be modulated by the rate of food flow at feeders and familiarity of food’s scent. Little is understood about how aversive foraging conditions such as predation and con-specific competition affect trophallaxis. We hypothesized that aversive conditions have an impact on food transfer inside the colony. Here we explore the effect of foragers’ aversive experience on downstream trophallaxis in a cage paradigm. Each cage contained one group of bees that was separated from feeders by mesh and allowed to feed only through trophallaxis, and another group that had access to feeders and self-specialized to either forage or distribute food. Our results show that aversive foraging conditions increase non-foragers’ trophallaxis with bees restricted from feeder access when food is scented, and have the opposite effect when food is unscented. We discuss potential behavioural mechanisms and implications for the impact of aversive conditions such as malaise inducing toxins, predation, and con-specific competition.

## Introduction

Collection of food is the driving force behind much of the activity within a eusocial insect colony. Sterile or functionally sterile workers are responsible for foraging as well as for distribution and processing of nutrients. In some species, behavioural specialization is morphologically fixed during development, while in other cases behavioural specialization can be modulated by social and environmental factors^1,2^. Whether roles change throughout an individual’s life or remain fixed, an essential facet of the resulting division of labour is that in any given moment a proportion of members do not forage and can experience external conditions only through a cascade of social interactions.

Honey bees, *Apis mellifera*, perform a wide array of behaviours inside and outside the nest. Individuals progress through various kinds of work throughout their lives in a progression that is partially determined by genetics and age but is also influenced by queen pheromone, brood pheromones, food availability, and season^3–5^. Some worker traits correlate specifically with behavioural role, such as circadian rhythm^6^, metabolism^7^, haemolymph levels of vitellogenin and juvenile hormone^8^, and expression of octopamine receptor *OA1* in the subesophageal ganglion and antennal lobes^9^. Other worker traits are better correlated with age rather than role, such as sucrose responsiveness^10^ and expression of octopamine receptor in the mushroom body^9^. Without a disruption of the age distribution in a colony, approximately 0–5 day old workers specialize in cleaning cells, ~3–12 day old bees act as nurses for developing brood, ~12–14 day old bees receive and store food, and older bees forage outside the hive^11^.

Trophallaxis, the mouth-to-mouth exchange of liquid, is a primary mechanism of food and appetitive information transfer inside a honey bee colony. The receiving bee places her proboscis between the mandibles of the donor and the two rapidly antennate as liquid is passed from donor to receiver^12^. Foraging conditions and the olfactory cues associated with incoming food influence the dynamics of ensuing trophallaxis. Nectar flow rate determines the rate at which foragers unload to a receiver, and subsequently the rate at which the first receiver unloads to the following receiver^13–15^. The presence of odour in nectar increases the frequency of trophallaxis events between waggle dances^16^. Unfamiliar odorants in the crops of donating foragers have been shown to reduce the occurrence of trophallaxis if a receiving forager has had prior appetitive olfactory experience^17^.

The process of collecting food involves a rich slew of sensory experiences, and can generate aversive experience as well as appetitive reward. Foragers are exposed to con-specific competition^18^, predation from a variety of arthropods^19^ as well as birds^20^, and to noxious secondary compounds excreted by various plants^21^. Aversive foraging experience causes foragers to avoid re-visitation of sites^22^ and to use the stop signal to inhibit recruitment of others to these sites^18^. The stop signal occurs when returning foragers identify dancing bees sharing samples of nectar that has an aversively associated scent, and vibrate against the dancer’s thorax to halt the dance^18,23^.

**Figure 1:**
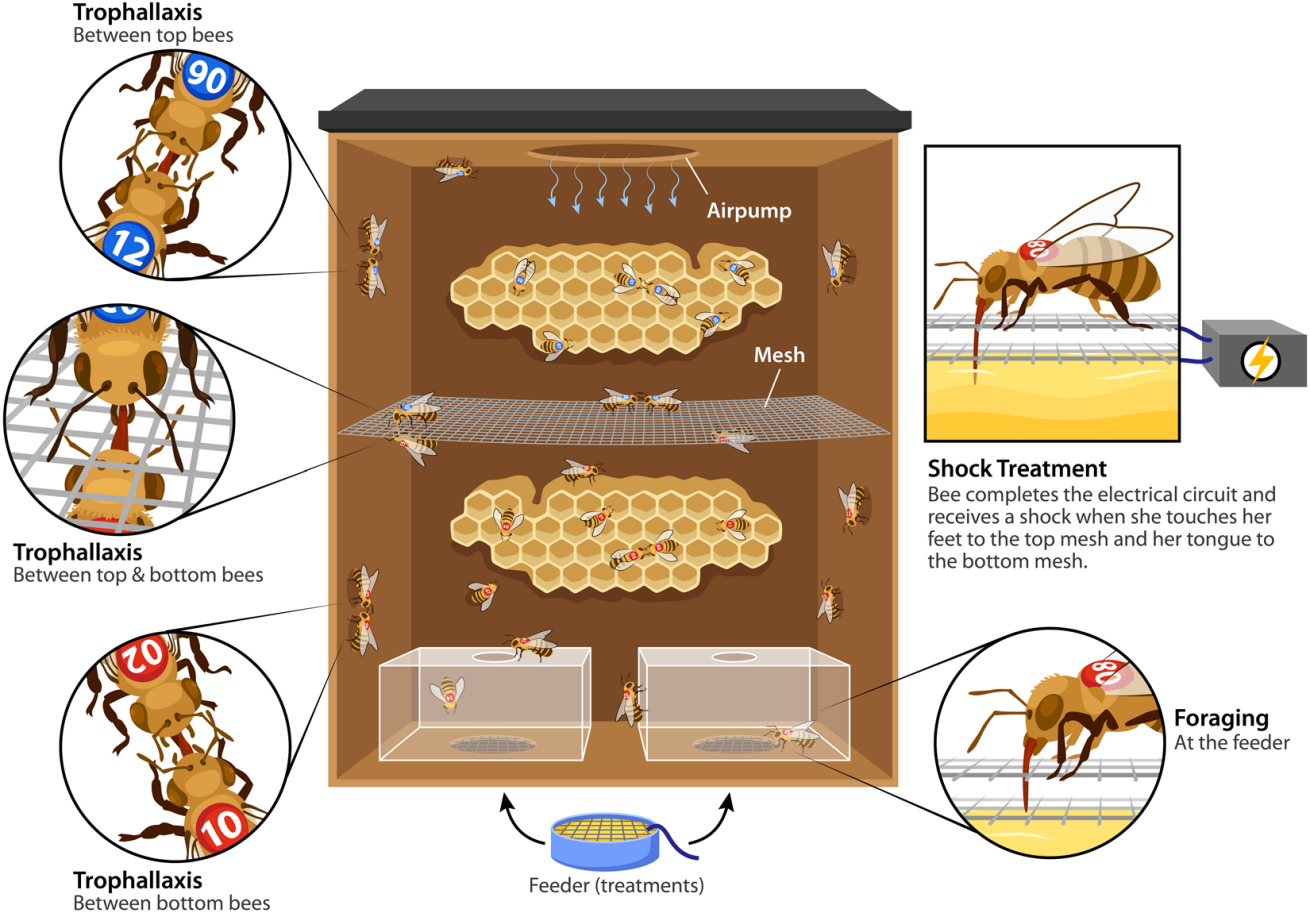
Cages provide four different foraging conditions. In a novel cage design, bees are separated into two compartments of which only the bottom allows foraging at feeders. Cages are 5″ 9/16 wide × 5″ 1/16 long × 2″ 3/8 deep. Bees in the top compartment are fed solely through trophallaxis. For the first 48 hours, cages provide different foraging conditions: 1. feeders offering sucrose. 2. feeders offering scented sucrose. 3. feeders offering sucrose paired with electric shock. 4. feeders offering scented sucrose paired with electric shock. Graphic illustration courtesy of Sabine Deviche and the Arizona State University Vislab. © 2018 Arizona State University. Used with permission.

Considering that foragers can communicate aspects of their foraging experience and that non-foraging receivers unload nectar to downstream bees at a rate positively correlated with the rate at which it was originally received, we were intrigued by the possibility that aversive foraging experiences could be transmitted to bees downstream the network of food sharing. The transfer of information regarding aversive foraging conditions to non-foragers could have the adaptive benefit of mitigating environmental risks once foraging is later initiated. As a first step toward understanding the relationship between foraging conditions and downstream social food transfer, we designed cages in which the top compartment held honey bees fed only through trophallaxis and the bottom compartment held bees with access to feeders offering different foraging conditions. Negative foraging conditions were simulated by feeders rigged with mild electric shock, which is the form of punishment used most commonly in the laboratory to explore bees’ aversive learning abilities and unconditioned aversive behavioural responses^24^. Nectar in the wild is sometimes scented and sometimes unscented^25,26^, so feeders offered either scented or unscented sucrose, creating an experimental design with four foraging conditions (see Fig. 1). To characterize different behavioural roles and the social feeding interactions between them, trophallaxis and foraging behaviours were recorded during short observation periods. Afterwards, long-term recall assays used the proboscis extension response to test bees’ preference for scents associated with different foraging conditions, to determine associations made either directly or through social means. Sucrose responsiveness and gene expression assays allowed us to explore inter-individual variation associated with the self-organized division of labour that emerged in cages.

## Results

### Self-specialization of feeding behaviour in cages

**Figure 2:**
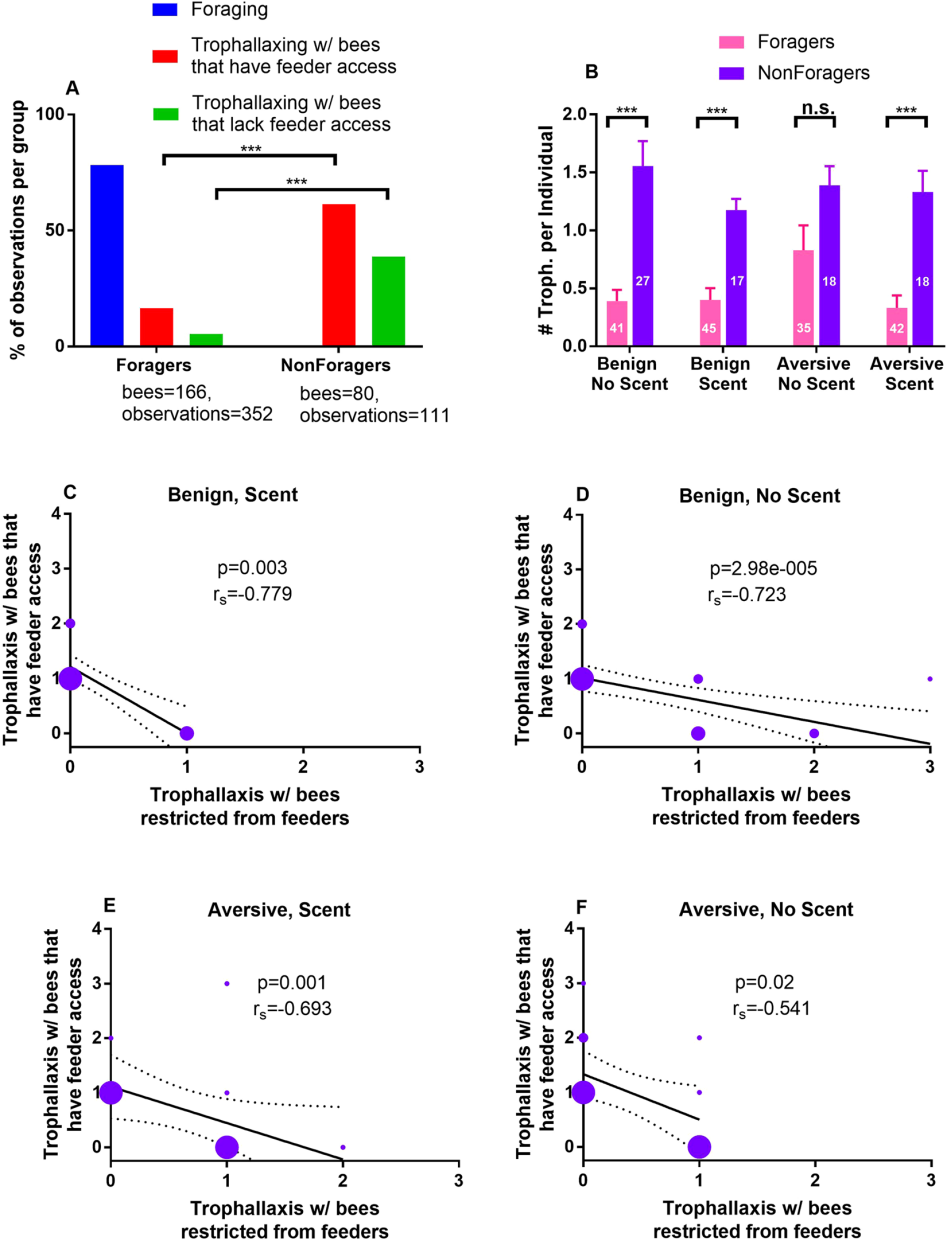
Caged bees self-organize into distinct behavioural roles. Foragers engage in trophallaxis less often than non-foragers. (**A**) Bars indicate percentages of total observed activity devoted to each behaviour for foragers and non-foragers. A significantly lower proportion of foragers’ overall activity is spent trophallaxing with bees that have feeder access (GLMM: p = 2.9e-07, Table 1A) as well as with bees restricted from feeders (GLMM: p = 6.6e-07, Table 1B). (**B**) Bars indicate the mean number of trophallaxis observations per individual, error bars show standard error of the mean. The difference in trophallaxis activity between foragers and non-foragers manifests in each of the different foraging conditions (Table 1C–F). (**C–F**) Bubble charts show the relation between two types of trophallaxis for non-foragers, with bubble size indicating the number of data points represented and dotted lines showing 95% confidence intervals of the linear regression line. Within the role of non-forager, there is a significant inverse correlation between trophallaxis with bees that have feeder access vs. bees restricted from feeders in each of the different foraging conditions.

We observed a self-organized division of labour in the bees with access to feeders. To characterize how the bees were collecting and distributing food, we spent a total of 1 hour recording instances of trophallaxis and foraging in each cage. Bees observed foraging at feeders at least once were defined as “foragers” and bees never observed foraging were defined as “non-foragers.” Overall, we found that foragers spent a smaller portion of their overall activity than non-foragers trophallaxing with other bees that had feeder access (GLMM: z = 4.97, p = 6.56e-07) (Table 1B) and with bees restricted from feeders (z = 5.13, p = 2.87e-07) (Table 1B) (Fig. 2a). Considering each of the four cage conditions separately, foragers spent less time than non-foragers engaged in trophallaxis; this distribution of behaviour failed to reach significance only when foraging conditions were aversive and food was scented (though the difference was still considerable in this case) (Table 1C–F, Fig. 2b). Although more than twice as many bees became foragers than non-foragers (166 and 80, respectively), the latter were responsible for 69% (n = 43 observations out of a total of 62) of all observed trophallaxis with bees restricted from feeders, and for 54% (n = 68 observations out of a total of 126) of all observed trophallaxis with bees that had feeder access.

**Table 1:** Zero-Inflated Generalized Linear Mixed Models – Effect of Foraging Role on Trophallaxis.

Fixed effects	Estimate	Std. Error	Z-value	Pr(>|z|)
**A**. **All conditions: trophallaxis with bees restricted from feeders**, **n = 246**				
AIC = 292 BIC = 306 deviance = 284 df. resid = 242 Random effect: Replicate, Variance = 0.0235, Std.Dev = 0.153				
Foraging Role	1.51	0.295	5.13	2.9e-07
**B**. **All conditions: trophallaxis with bees that have feeder access**, **n = 246**				
AIC = 451 BIC = 465 deviance = 443 df. resid = 242 Random effects: Replicate, Variance = 6.24e-11, Std.Dev = 7.9e06				
Foraging Role	0.889	0.179	4.97	6.6e-07
**C**. **Non-scented food**, **benign conditions: overall trophallaxis**, **n = 68**				
AIC = 150.4 BIC = 159.3 deviance = 142.4 df. resid = 64 Random effects: Replicate, Variance = 1.09e-19, Std.Dev = 1.05e-05				
Foraging Role	−1.383	0.294	−4.71	2.5e-06
**D**. **Non-scented food**, **aversive conditions: overall trophallaxis**, **n = 56**				
AIC = 151.9 BIC = 160 deviance = 143.9 df. resid = 52 Random effects: Replicate, Variance = 0.0681, Std.Dev = 0.261				
Foraging Role	0.554	0.288	1.92	0.055
**E**. **Scented food**, **benign conditions: overall trophallaxis**, **n = 62**				
AIC = 121 BIC = 129.5 deviance = 113 df. resid = 58 Random effects: Replicate, Variance = 4.02e-11, Std.Dev = 6.34e-06				
Foraging Role	1.079	0.325	3.32	9e-04
**F**. **Scented food**, **aversive conditions: overall trophallaxis**, **n = 60**				
AIC = 117.8 BIC = 126.2 deviance = 109.8 df. resid = 56 Random effects: Replicate, Variance = 1.47e-10, Std.Dev = 1.21e-05				
Foraging Role	1.386	0.336	4.12	3.8e-05

Non-foragers further specialized to produce a strong inverse correlation between trophallaxis with bees in their own compartment and trophallaxis with bees restricted from feeders, in each of the four foraging conditions (Spearman Rank Correlations: benign, non-scented (n = 26) − r_s_ = −0.723, p = 2.98e-005; benign, scented (n = 17) − r_s_ = −0.779, p = 0.003; aversive, scented (n = 18) − r_s_ = −0.693, p = 0.001; aversive, non-scented (n = 18) − r_s_ = −0.541, p = 0.02) (Fig. 2c–f).

### Relationship between sucrose responsiveness and food sharing behaviour

**Figure 3:**
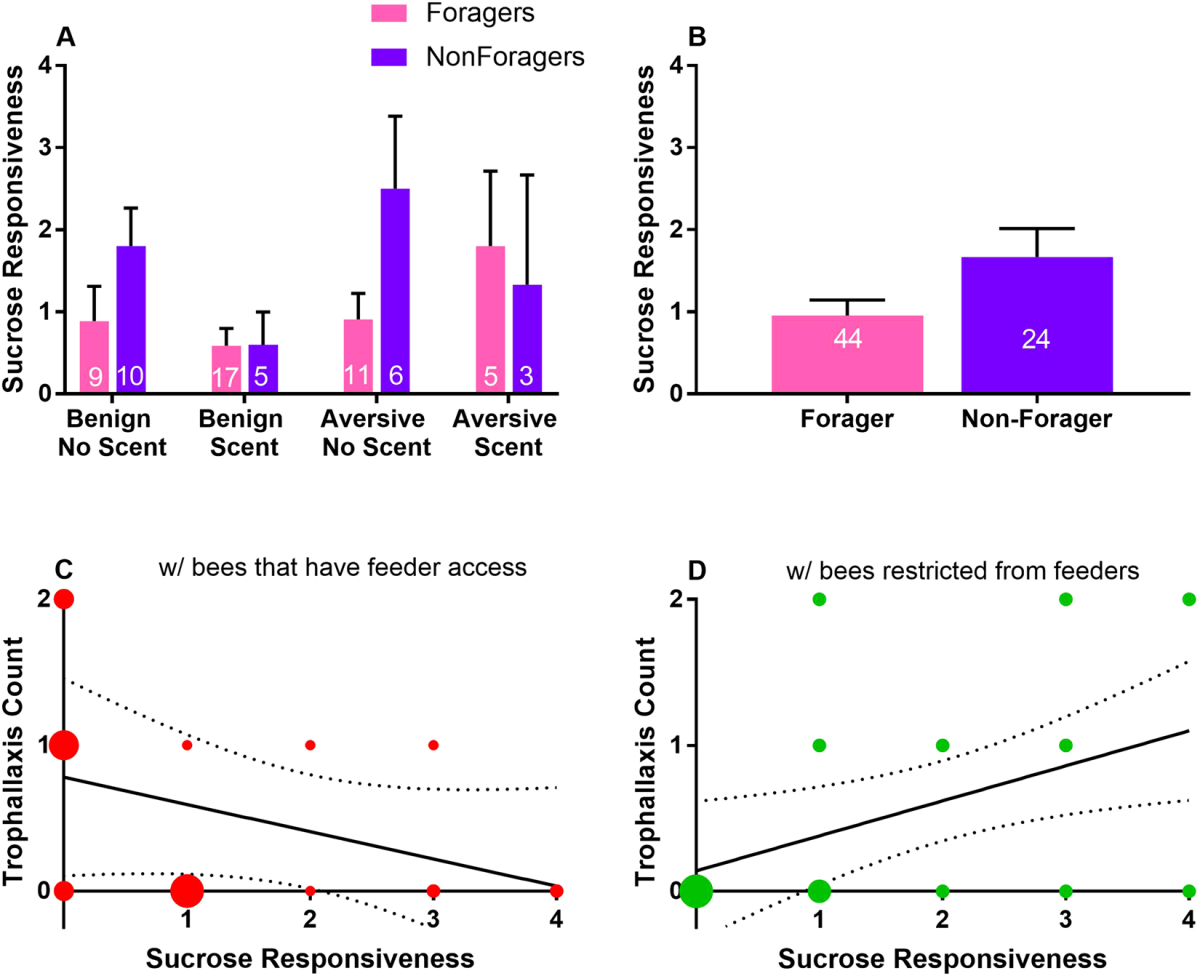
Relationship between sucrose responsiveness and food sharing behaviour. Bars show the mean sucrose responsiveness scores of foragers vs non-foragers, error bars show standard error of the mean. Bubble size indicates the number of data points represented and dotted lines show 95% confidence intervals of the linear regression line. There is no significant difference in sucrose responsiveness between foragers and non-foragers in any of the foraging conditions (**A)** or when sucrose responsiveness scores from all conditions are pooled (**B)**. In benign foraging conditions when food is unscented, sucrose responsiveness is (**C)** inversely related to trophallaxis with bees that have feeder access (Spearman’s rank order correlation: n = 19, r = 0.523, p = 0.022) and **(D)** positively related to trophallaxis with bees restricted from feeders (n = 19, r = −0.46, p = 0.048).

Sucrose responsiveness has classically been studied in relation to foraging behaviour^10,27,28^ and has not been considered in the context of social food sharing. We find no difference in the sucrose responsiveness of caged foragers vs. non-foragers (Mann Whitney U Tests: overall (non-forager n = 24, forager n = 44), U = 408, p = 0.105; benign, no scent (non-forager n = 9, forager n = 10), U = 29.5, p = 0.202; benign, scent (non-forager n = 5, forager n = 17), U = 41.5, p = 0.999; aversive, no scent (non-forager n = 6, forager n = 11), U = 18, p = 0.124; aversive, scent (non-forager n = 3, forager n = 5), U = 6, p = 0.821) (Fig. 3a,b). However, in benign conditions with non-scented sucrose, sucrose responsiveness is positively related to trophallaxis with bees restricted from feeders (Spearman Rank Correlations: n = 19, r_s_ = 0.523, p = 0.022) and inversely related to trophallaxis with bees that have feeder access (n = 19), r_s_ = −0.46, p = 0.048) (Fig. 3c,d). The sucrose responsiveness data is part of a larger dataset used for different analyses in [Finkelstein *et al*., under review at Scientific Reports].

### *AmOctαR1* expression and foraging behaviour

**Figure 4:**
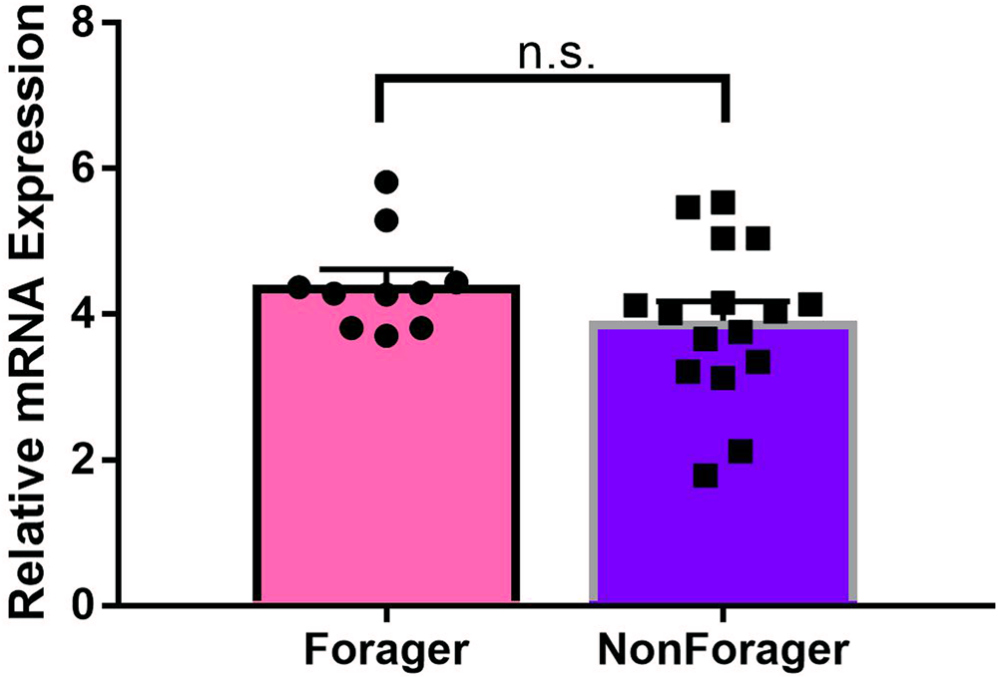
Subesophageal ganglion expression of *AmOA1* for different foraging roles. Bars show the mean receptor expression levels for foragers vs non-foragers, error bars show standard error of the mean. There is no difference in subesophageal ganglion expression levels of *AmOA1* between foragers and non-foragers (Student’s T Test, two-tailed: n (foragers) = 16 and n (non-foragers) = 10, t = 0.32, p = 0.201).

Free-flying age-matched foragers and nurses differ in expression levels of octopamine receptor *AmOctαR1* in subesophageal ganglion and antennal lobes^9^. However, we did not find a difference in subsesphageal ganglion expression levels for the caged bees (Student’s t test, double-sided, foragers n = 10, non-foragers n = 16, t = 1.32, df = 24, p = 0.201) (Fig. 4). Data is a subset of dataset used for different analyses in [Finkelstein *et al*., under review at Scientific Reports].

### Effect of foraging conditions on foraging and social food sharing

**Figure 5:**
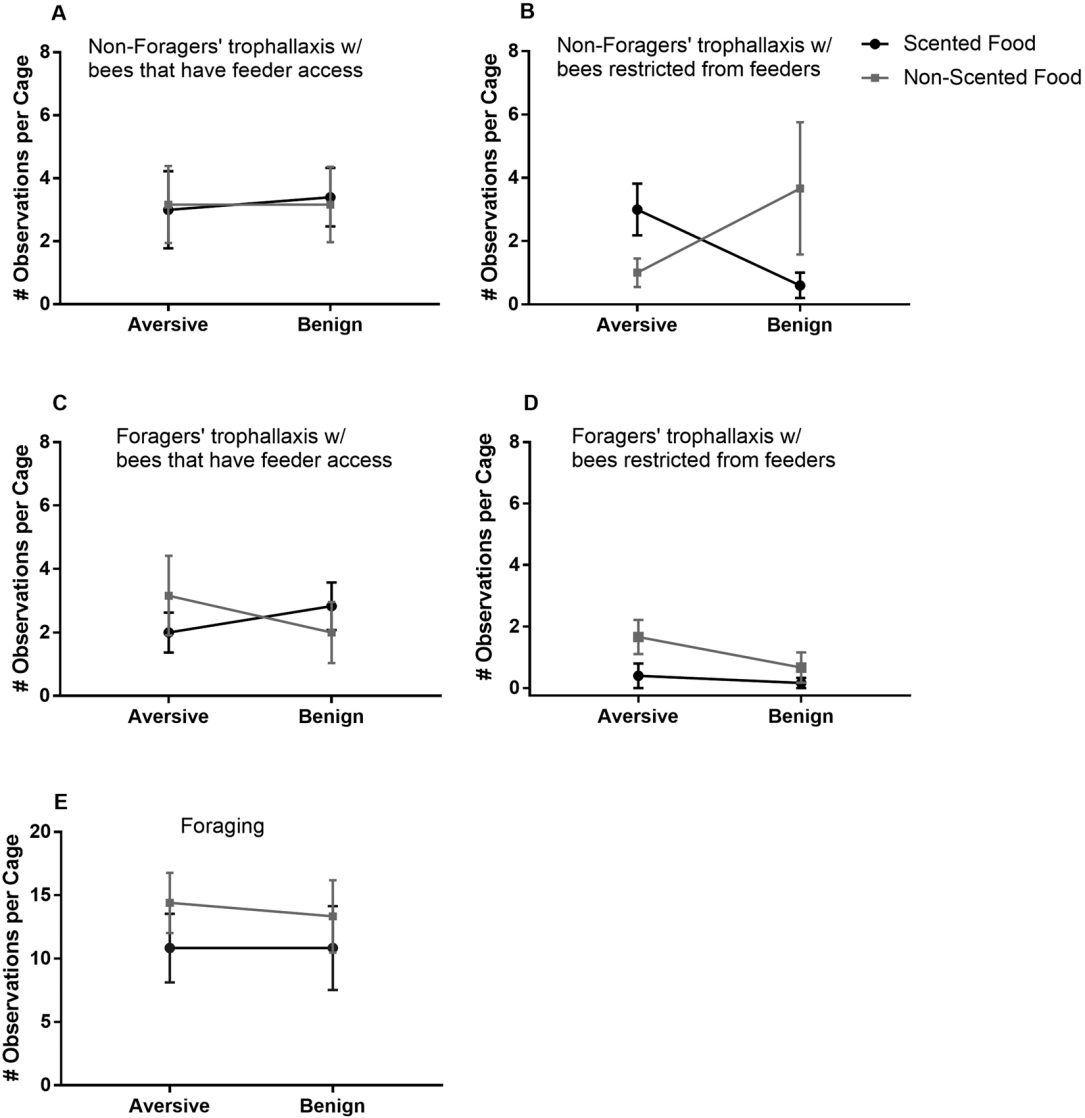
Influence of foraging conditions on trophallaxis dynamics. Interaction charts show mean numbers of observed trophallaxis events in cages with different foraging conditions. Error bars show standard error of the mean. The only significant effects occur for non-foragers’ trophallaxis with bees restricted from feeders (**B**). When food is scented, aversive conditions increase the number of trophallaxis events with bees restricted from feeders. For unscented food, aversive conditions have the opposite effect (Table 2A).

To determine the effect of foraging conditions on downstream social food sharing, we recorded all trophallaxis events that occurred within two 30 minute observation periods. A Poisson Generalized Linear Model with a random effect of cage showed that the foraging conditions significantly impacted only non-foragers’ trophallaxis with bees restricted from feeders (p = 6.34e-06 for interaction of scent and aversive conditions, p = 0.00139 for scent, p = 0.00283 for aversive conditions) (Table 2A). When food was scented, aversive conditions increased trophallaxis with bees restricted from feeders; when food was unscented, aversive conditions had the opposite effect (Fig. 5b). All other types of food sharing behaviour, and foraging frequency, were not influenced by foraging conditions (Fig. 5a,c,d and Table 2B–F).

**Table 2:** Generalized Linear Mixed Models – Effects of Foraging Conditions on Foraging and Trophallaxis.

Fixed effects	Estimate	Std. Error	Z-value	Pr(>|z|)
**A**. **Non-foragers’ trophallaxis with bees restricted from feeders**, **n = 27**				
AIC = 97.4 BIC = 103.8 deviance = 87.4 df. resid = 22 Random effect: Replicate, Variance = 0.6687, Std.Dev = 0.8177				
Scent	−1.91	0.597	−3.20	0.00139
Aversive Conditions	−1.30	0.435	−2.99	0.00283
Scent:Aversive Conditions	3.63	0.803	4.51	6.34e-06
**B**. **Non-foragers’ trophallaxis with bees that have feeder access**, **n = 27**				
AIC = 138.2 BIC = 144.6 deviance = 128.2 df. resid = 22 Random effects: Replicate, Variance = 0.01702, Std.Dev = 0.1305				
Scent	0.054	0.292	0.186	0.853
Aversive Conditions	−0.026	0.280	−0.093	0.926
Scent:Aversive Conditions	−0.099	0.472	−0.209	0.834
**C**. **Foragers’ trophallaxis with bees restricted from feeders**, **n = 23**				
AIC = 58.4 BIC = 64.0 deviance = 48.4 df. resid = 18 Random effects: Replicate, Variance = 0.01221, Std.Dev = 0.1105				
Scent	−1.39	1.19	−1.24	0.215
Aversive Conditions	0.916	0.592	1.55	0.121
Scent:Aversive Conditions	−0.044	1.36	−0.033	0.974
**D**. **Foragers’ trophallaxis with bees that have feeder access**, **n = 23**				
AIC = 104.0 BIC = 109.7 deviance = 94.9 df. resid = 18 Random effects: Replicate, Variance = 0.167, Std.Dev = 0.4087				
Scent	0.348	0.377	0.924	0.356
Aversive Conditions	0.459	0.369	1.25	0.213
Scent:Aversive Conditions	−0.884	0.545	−1.62	0.105
**E**. **Bees restricted from feeders trophallaxis with others in same compartment**, **n = 23**				
AIC = 385.7 BIC = 391.4 deviance = 375.7 df. resid = 18 Random effects: Replicate, Variance = 0.138, Std.Dev = 0.3715				
Scent	−0.177	0.137	−1.29	0.196
Aversive Conditions	0.066	0.129	0.514	0.607
Scent:Aversive Conditions	0.284	0.197	1.44	0.149
**F**. **Foraging**, **n = 23**				
AIC = 162.1 BIC = 167.8 deviance = 152.1 df. resid = 18 Random effects: Replicate, Variance = 0.1935, Std.Dev = 0.4399				
Scent	2.07e-01	1.67e-01	1.24	0.214
Aversive Conditions	1.58e-08	1.75e-01	0.00	1.00
Scent:Aversive Conditions	−3.47e-02	2.39e-01	−0.145	0.885

### Long-term influence of socially transferred food on scent preference

**Figure 6:**
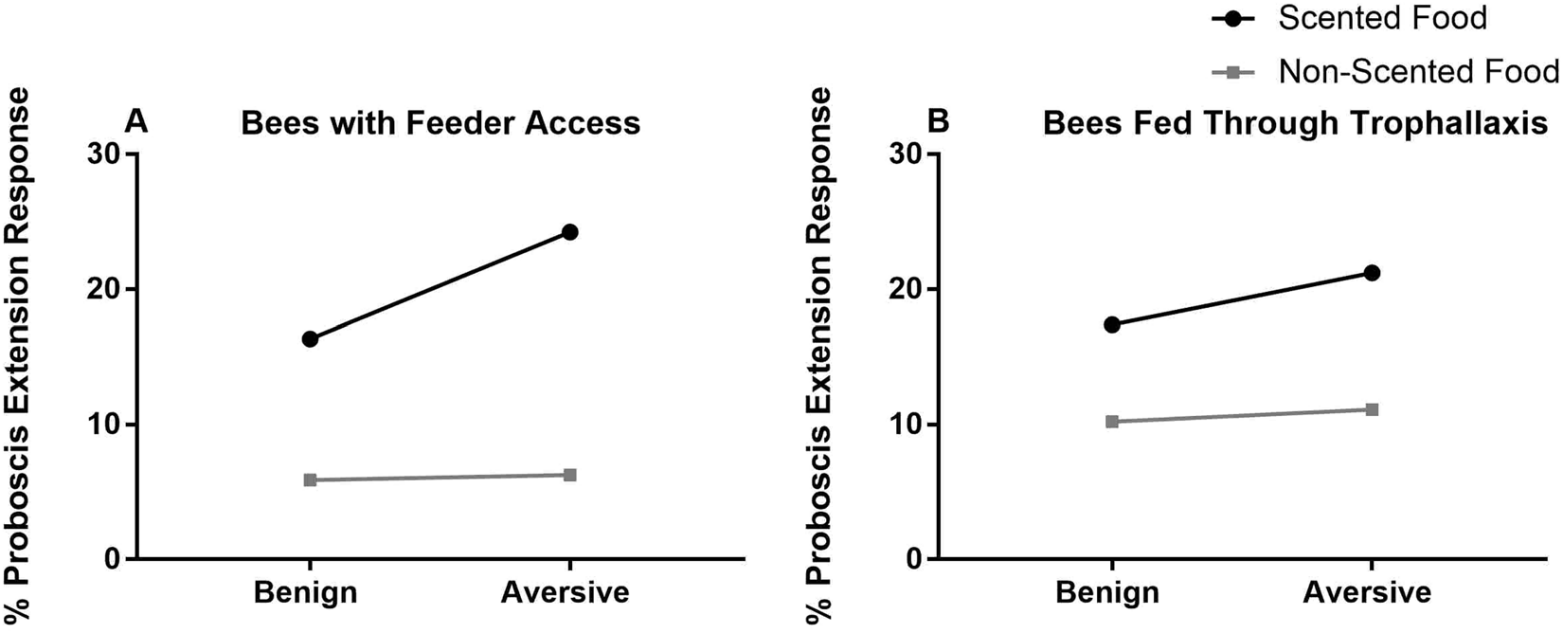
Long-term recall of scent dissolved in food in different foraging conditions. Line charts show percentages of bees that responded with proboscis extension to the scent used in cages and did not respond to a novel scent. (**A**) Bees with access to feeders exhibit significant long-term recall of scent with no effect of aversive foraging conditions. (**B**) Bees restricted from feeders do not exhibit significant long-term recall of scent. (See results for details).

Long-term recall tests were used to determine the effect of scented food collected at benign and aversive feeders on subsequent foraging preferences, both for bees that had access to feeders and bees fed through trophallaxis. Tests consisted of randomized presentations of a novel scent and the scent that been used in cages. Bees that had feeder access in cages with scented food exhibited recall 3 days later, regardless of whether the scented food had been paired with aversive electric shock (Logistic Generalized Estimating Equation, N = 181, for predictor “scent:” β1 Wald χ^2^ = 6.87, p = 0.0088. For predictor “electric shock:” β1 Wald χ^2^ = 0.28, p = 0.6) (Fig. 6a). Bees in the same cages that did not have feeder access and were fed through trophallaxis did not exhibit significant long-term recall, although this could have been due to overall low PER (Logistic Generalized Estimating Equation, N = 182, for predictor “scent:” β1 Wald χ^2^ = 2.46, p = 0.117) (Fig. 6b). Dataset also used to address a different question in [Finkelstein *et al*., under review at Scientific Reports].

## Discussion

Our cage paradigm gives rise to a self-specialized dynamic of food distribution similar to what is observed in honey bee colonies. Natural foragers returning to the nest unload their crop to a single non-foraging receiving bee, only sharing with additional receivers if the crop load is more than can be imbibed by just one^29^. The receiving bee trophallaxes the nectar to other hive mates or deposits it in honey comb^30^. Correspondingly, caged individuals that were observed to forage at least once were seldom observed to share food with others, while bees that also had access to feeders but were never observed to forage were far more likely to engage in trophallaxis (Fig. 2a,b). For simplicity, we will henceforth refer to the first group as “foragers” and to the latter as “non-foragers.” Within the group of non-foragers, there was an inverse correlation between trophallaxis with bees restricted from feeders and trophallaxis with bees that had feeder access (Fig. 2c,f), suggesting a further subdivision of food sharing roles. These patterns indicate a cascade of trophallaxis in which foragers unload food to non-foragers specializing in receiving and distributing it to downstream non-foragers which in turn transfer food to the bees behind mesh.

In a natural hive, roles associated with food collection and distribution are marked by behavioural and physiological differences. Octopamine receptor *AmOctαR1* is differentially expressed in the antennal lobes and subesophageal ganglion of same-aged foragers and non-foragers^9^. Sucrose responsiveness tends to increase with age; earlier work shows a difference between same-aged nurses and foragers^10^, but this was not measurable in a later study^28^. Although sucrose responsiveness has been often studied in relation to foraging behaviour, it has not previously been examined in the context of food sharing behaviours. To better understand the self-specialization that emerged inside cages, we quantified sucrose responsiveness as well as expression levels of *AmOctαR1*. Our results do not show differences in *AmOctαR1* expression between foragers and non-foragers, suggesting that this is not necessary for the determination of foraging role in an artificial caged laboratory setting (Fig. 4). Similarly to the most recent findings in outdoor colonies^28^, we find no difference in sucrose responsiveness between foragers and non-foragers (Fig. 3a,b). However, in benign foraging conditions when food is unscented, we find that sucrose responsiveness is inversely related to trophallaxis with bees that have feeder access and positively correlated to trophallaxis with bees restricted from feeders (Fig. 3c,d). Aversive conditions and scent negate the correlations between trophallaxis and sucrose responsiveness. In the unscented benign condition, these opposite relationships with sucrose responsiveness could be driving the specialization for distributing food within the bottom compartment vs transferring food to bees restricted from feeders (Fig. 2c,f). Our observations did not reliably identify directionality of food transfer, so it is unclear whether the relationships with sucrose responsiveness are specific to donating food or to receiving it. Future studies will be necessary to resolve the causality of the relationship between food sharing and sucrose responsiveness.

Surprisingly, we find that foraging conditions and presence of scent change only the food sharing behaviour of non-foragers that were never observed directly experiencing the aversive conditions (Fig. 5). When food is scented, non-foragers in cages with aversive foraging conditions were more likely to trophallax with bees restricted from feeder access, without any difference in trophallaxis with bees that had feeder access. When food is unscented, aversive foraging conditions have the opposite effect, decreasing trophallaxis with bees restricted from feeder access. It is important to note that no other trophallaxis or foraging behaviours are altered, suggesting a specific effect on a particular type of social interaction rather than a global effect of stress (Fig. 5a,c–e). The dependence of the effect of aversive conditions on scent is ecologically relevant, as nectar in the wild is sometimes but not always scented^25,26,31^. The corresponding behaviour in a natural colony would be an increase or decrease in trophallaxis between receiver bees and younger nurse bees.

An inhibition of trophallaxis with younger nurse bees under threatening conditions aligns with the phenomenon in which some species of birds decrease their parental provisioning to redirect energy towards self-maintenance in stressful conditions^32,33^. On the evolutionary scale, unpredictable and poor environments lead to parents paying less attention to chicks’ begging and instead using cues such as body size to determine provisioning^34^. Aversive foraging conditions could have similar consequences for a honey bee colony; whether non-foragers experience stress through an unobserved direct encounter with aversive feeders or the stress is communicated via social interaction with foragers, this results in less social feeding of the bees lacking feeder access. Future studies could use observation hives to test our prediction that receivers trophallax unscented nectar less to young bees in aversive foraging conditions. More broadly, our results give rise to the question of whether food is shared less with the youngest members of a social group in times of environmental stress across vertebrate and invertebrate species besides birds and honey bees.

The presence of scent in food provides an adaptive opportunity to pass on information regarding danger. If trophallaxis between non-foragers and downstream bees can transmit a negative association with the nectar’s scent, future risk will be mitigated once the downstream bees initiate their own foraging decisions. Researchers have long known that trophallaxis likely serves communication purposes beyond the sharing of food; when trophallactic interactions in small groups of bees were studied, less than 5% of the interactions actually resulted in food transfer^12^. Social mammals such as rats^35^, mice^36^, Mongolian gerbils^36,37^, and spiny mice^38^ confer long-lasting food preference in conspecifics using semiochemicals found on the breath and in urine and faeces. Negative feedback that transmits food source aversion is less common. *Lasius niger* ants deposit less trail pheromone when a trail is crowded, thus creating a reduction in positive signal that serves as a mechanism of negative feedback^39^. Food receivers in colonies of the stingless bee *Melipona seminigra* food are vibrated on their thorax by foragers during unloading^40^, and in *Melipona quadrifasciata* scented food elicits stronger vibrations than unscented food during unloading^41^; the effect on receivers has not yet been discovered. Honey bee foragers that have had an aversive experience at a food source identity other foragers dancing to advertise a similarly scented location, and stop the dance with a vibrational “stop signal” to reduce recruitment^18^. In a laboratory setting, honey bees produce a hissing sound when presented with an odour they have learned to associate with electric shock^42^. It is not yet clear whether this hiss is similarly produced in the natural context of a hive and whether it acts as a social signal. Both the vibrational stop signal and the hiss are activated by aversively associated scents, providing a potential mechanism for how aversive conditions impact downstream bees’ behaviour when food is scented. One possibility is that foragers and/or non-foragers sense aversively associated scents while unloading scented sucrose from their own crops, which triggers a hiss, vibration, or some other informative social behaviour. We occasionally observed bees running at higher than usual speeds inside cages and vibrating against other bees, but it was not possible to identify the bees’ tag numbers during such rapid movement and video recording equipment was not used. It was also not possible to hear individual hisses through the plastic window covering cages due to interference from the air pump and the background noise of bees’ buzzing. Although further work is needed to reveal the mechanisms at play, our results suggest that aversively associated scented nectars not long increase non-foragers’ trophallaxis with downstream bees but also facilitate the transfer of information regarding nectar sources.

In order for an increase in trophallaxis of food collected in aversive conditions to be adaptive, downstream bees must exhibit subsequent aversion to the associated scent. This is particularly essential because foragers generally prefer food sources that smell like what they have been fed through trophallaxis earlier in their lives. Bees as young as a few days post- emergence are primed by the scented nectars foragers bring into the nest, forming positive associative memories that can be retrieved once they reach foraging age^43^. Adult bees prefer flowers smelling of nectars they have consumed in the nest^16^. Similarly, *Camponotus mus* ants that have received scented solution in a single trophallaxis event prefer this scent when tested in a Y-maze^44^. Thus, one might expect the maladaptive consequence of increased trophallaxis causing preference for riskier food sources unless the aversive association continues to be transferred through trophallaxis. After removing bees from cages, we tested all subjects’ proboscis extension responses to the scents that had been dissolved in food at aversive and benign feeders. We predicted that both bees with and without feeder access would exhibit proboscis extension to scents from benign feeders and not to scents from aversive feeders. However, aversive conditions had no effect on proboscis extension for bees in either compartment (Fig. 6). Although bees with feeder access did exhibit statistically significant preferential PER to the scent experienced in cages, regardless of aversive conditions, <30% of these bees displayed PER, suggesting that due to the accumulated stress of five days in cages followed by one day of restraint in harnesses, bees may not have been able to display PER even in the case of preference. For bees without feeder access, no significant preference was detected even in benign conditions, so the impact of aversive conditions was not clear. We hope that future studies using free-flying, less stressed subjects can test the idea that aversive conditions cause receiver bees to not only increase their rate of trophallaxis with nest bees, but also to pass on the aversive association, thus preparing as many bees as possible for dangerous food sources.

This study suggests that aversive conditions experienced during foraging can change downstream social food sharing, influencing pre-foraging individuals that have not yet experienced external conditions first-hand. We hope to spur future work exploring the impact of natural aversive foraging conditions, such as predation and con-specific competition, on social information transfer and subsequent foraging decisions.

## Materials and Methods

### Ethical note

There were no environmental implications of the experimental procedures, as honey bees were collected from laboratory colonies on campus and brought into the laboratory for experiments. To simulate aversive foraging conditions with electric shock, the minimum effective voltage was used; 4.2 V administered as described in Fig. 1 induced a brief initial startle response but no avoidance. A total of ~800 female, pre-foraging aged *Apis mellifera ligustica* bees were used.

### Bee collection and cage setup

In order to collect developmentally mature honey bees without prior foraging experience, individuals found on frames containing honey and no brood were first touched lightly with forceps to cause foragers to fly off. For each replicate, bees were collected from three different hives and mixed to prevent hive effects. Bees were immobilized in glass vials on ice, labelled with a queen bee number tag (The Bee Works Queen Marking Kit, Ontario, Canada) on the thorax, and placed inside an experimental cage (Fig. 1) in which they regained mobility. Each cage was divided by mesh to separate bees into two groups: 17 bees with feeder access in the bottom compartment and 13 bees with no feeder access in the top compartment (Fig. 1). Empty honey comb lined the back wall in both compartments. The cages were kept in constant light conditions at room temperature (20–26 ^o^C). Cage locations within the experimental room were alternated.

Six replicates were run at Arizona State University in Tempe, Arizona from April – June 2015. Each replicate consisted of four different foraging conditions in four separate cages. For the first two days, feeders contained a cap from a 50 ml conical Eppendorf tube (Eppendorf, Hamburg, Germany) filled with 2 Kimwipes (Kimberly-Clark Professional, Roswell, Georgia, USA) saturated with 1.5 M sucrose. In two of the four cages, sucrose was scented with the floral scent linalool or phenylacetaldehyde (Sigma Aldrich, St. Louis, MO, USA); 50ul odorant per L of 1.5 M solution (Farina *et al*. 2007). One cage with unscented sucrose and one cage with scented sucrose provided aversive foraging conditions by delivering 4.2 V of electric shock to bees as they foraged at feeders (Fig. 1). Two feeder slots at the bottom of each cage allowed food location to be alternated every 6–8 h, thus reducing spatial learning of food location and limiting learning to the olfactory stimulus reliably paired with food.

After two days of treatment, the feeder slots at the bottom of all boxes were cleaned with a Kimwipe dampened with ethanol to remove traces of sucrose, odour, or honey bee secretions. For three days, both feeder slots in all cages were filled with Eppendorf tube caps containing two Kimwipes soaked in 0.5 M sucrose. These were replaced to maintain constant saturation.

### Trophallaxis and foraging observations

Each cage was observed for a total of one hour, 30 min per day, for the two days of differential foraging conditions. The time of day and observer (either A. B. Finkelstein or one of two undergraduate students) were alternated between foraging conditions. The observer recorded instances of foraging from feeders, trophallaxis between forager bees, trophallaxis between bees in the bottom and top compartments, and trophallaxis between bees in the top compartment. Foraging was recorded only when a bee’s proboscis was observed touching the feeder surface, and trophallaxis was recorded when one or several bees’ proboscises were placed between another bee’s mandibles. Bees were identified by the coloured number tag on their thoraxes.

### Preparation for behavioural assays

A randomly selected majority of bees were chilled on ice to be individually harnessed, while a smaller portion of bees was flash frozen in liquid nitrogen for a separate study. Bees to be used for behavioural assays were allowed collected from cages into glass vials, chilled on ice until immobile, and restrained in custom metal harnesses with strips of duct tape^45^. After acclimating for 30 min, bees that had been restricted from feeder access were fed with 2 μl of 0.5 M sucrose to prevent starvation, as they may have been less sated than the bees with *ad libitum* access to sucrose.

### Behavioural assays

#### Long-term recall tests

After 3 hrs of acclimatization (3 days after cessation of treatment), all bees were tested for long-term recall of the scent experienced in cages offering scented sucrose (phenylacetaldehyde or linalool). One of the following scents were used as a novel control during test trials; 2-octanol or 2-hexanol (Sigma Aldrich, St. Louis, MO, USA), both of which have been used in previous works studying olfactory memory and perception^46,47^. Odor cartridges built from a glass 1 cc tuberculin syringe barrel (BD Medical, Franklin Lakes, NJ) and a short length of silicon tubing (Cole-Parmer, Vernon Hills, IL) were used to deliver streams of scented air. 10 μl of pure odour was spread on a strip of filter paper (Whatman 114, Sigma-Aldrich, St. Louis, MO) that was pushed into the cartridge. Tubing attached the narrow end of odour cartridges to the automated odour delivery system, so that the wide end rested approximately 2 cm from bees’ antennae. A DirectLogic 05 programmable logic controller (Automation-Direct, Cumming, GA) was used to coordinate scent delivery by opening a valve (The Lee Co., Westbrook, CT) that allowed an airstream (~400 ml/min) to pass through the cartridge containing scented filter paper for 4 seconds. To clear the scent from the testing area between trials, a continuous flow exhaust system was placed ~5 cm behind the bee. Each bee was presented once with whichever scent had been used in the cages with scented sucrose (linalool or phenylacetaldehyde) and once with a novel scent (2-octanol or 2-hexanol) in a pseudo-random order, with at least 10 minutes between trials. Bees’ responses to each scent were recorded as a binary “yes” or “no,” with “yes” indicating that bees exhibited a proboscis extension reflex (PER), extension of the proboscis beyond an imaginary line between the opened mandibles, during scent presentation^45^. Afterward, all bees were allowed to feed on 0.5 M sucrose until sated. Bees were considered sated when touching the antennae with a droplet of sucrose did not induce PER.

#### Sucrose responsiveness

The following day, 96 h after the cessation of different foraging conditions and 12 h after all bees had been sated, sucrose responsiveness was determined by stimulating the antennae of randomly ordered harnessed bees with ascending concentrations of sucrose^48^. Sequentially presented droplets of 0.1%, 0.3%, 1%, 3%, 10%, 30% sucrose were interspersed with water between each trial to reduce and control for sensitization. The inter-stimulus interval for each bee was at least 10 minutes. Both antennae were touched with the droplet of sucrose or water, and the Proboscis Extension Responses were recorded to produce a Sucrose Responsiveness Score (SR) of 0–6. Bees that responded to water in between trials were excluded from subsequent analyses, as their responses to sucrose might have been due to water responsiveness.

#### Learning novel odours

Four hours after sucrose responsiveness assays, bees were tested on an olfactory discrimination assay for another study before being flash frozen for gene expression analysis. Throughout this assay, bees exhibited signs of poor health likely due to the time spent in cages followed by two days spent in harnesses; drooping heads, slightly extended proboscises, and lack of response to the 1.5 M sucrose solution.

### Octopamine receptor gene expression analysis

To investigate octopamine *AmOctαR1* receptor expression in foragers vs non-foragers, bees were flash frozen in liquid nitrogen after behavioural assays. Subesophageal ganglion neuropiles were subsequently dissected and individually stored. Only bees from cages offering benign foraging conditions were used in order to exclude any additional effect of aversive experience. RNA was extracted using a trizol/chloroform protocol (ThermoFisher Scientific, Waltham, MA, USA). The RNase-Free DNase set was used to remove DNA (Qiagen, Hilden, Germany) before a NanoDrop™ 2000/2000c Spectrophotometer (ThermoFisher Scientific, Waltham, MA, USA) determined RNA concentrations and defined purity with 260/280 ratios ranging from 1.8 to 2. 100 ng total RNA was used per reaction for cDNA synthesis for each sample with the Taqman Reverse Transcription Reagents Kit (ThermoFisher Scientific, Waltham, MA, USA). Established *AmOctαR1* primers^9^ were used to perform quantitative real-time Polymerase Chain Reaction using the ABI PRISM® 7000 Sequence Detection System (ThermoFisher Scientific, Waltham, MA, USA) to compare expression levels between foragers and non-foragers. *Elongation factor 1 alpha* was the reference gene used for normalization of the receptor transcripts as in full colonies it is stably expressed in nurse bees and foragers^9^. Three technical duplicates were used for each biological sample, and a negative control with no biological sample was included for each master mix. The same fluorescence threshold was set to determine the quantification cycle values for both genes, and the mean of the triplicate for each cDNA sample was used.

### Statistics

Statistical analyses and figures were completed in R Version 3.5.1^49^ and GraphPad Prism Version 7.00^50^.

Bees’ responses in the odour long-term recall test were scored as binary response (PER was scored as 1, no response as 0). These data were analysed with logistic generalized estimating equations using the binomial error structure with the logit-link function, a recommended way to compare categorical data^51^. The dependent variable “response to only cage scent” was scored as “1” if bees exhibited PER to the scent used in cages with scented sucrose and “0” if bees exhibited PER to both this and the novel scent or to no scents. The interaction term of “scent x electric shock” was included in analyses, and was removed from the model if it was not shown to be significant.

Self-specialization of foragers vs non-foragers was characterized using the total number of observations of each social feeding behaviour per individual bee. Many bees were never observed to trophallax, leading to a zero-enriched dataset. We therefore used a zero-inflated Generalized Linear Mixed Model (ziGLMM) using a Poisson distribution^52^ with a random effect of “cage” to compare individual trophallax counts. The ziGLMM determined the effect of “foraging role” on trophallaxis with bees that had feeder access and trophallaxis with bees restricted from feeders

To compare social dynamics, we used the total numbers of times bees were observed performing each behaviour per cage (foraging, trophallaxing with a bee in the same compartment, and trophallaxing with a bee in the other compartment). Two bees trophallaxing in the same compartment were therefore recorded as two instances of a bee trophallaxing with another bee in the same compartment. A Generalized Linear Mixed Model using a Poisson distribution with a random effect of “cage” quantified the main and interaction effects of scent and aversive conditions^52^.

Surose responsiveness scores (SR) did not follow a normal distribution, therefore we used the two-tailed Mann Whitney U Test to compare foragers and non-foragers, and Spearman Rank Order Correlations to identify correlative relationships with trophallaxis. Octopamine receptor expression passed a normality test for both groups (D’Agostino & Pearson normality test, for foragers K2 = 4.22, p = 0.121 and for non-foragers K2 = 0.4228, p = 0.908) so the parametric Student’s two-tailed *t*-test was used to compare expression levels between groups.

## Electronic supplementary material

Dataset 1

## Data Availability

All data generated or analysed during this study are included in this published article (and its Supplementary Information files).

## References

[1] Mertl AL, Traniello JFA (2009). Behavioral evolution in the major worker subcaste of twig-nesting Pheidole (Hymenoptera: Formicidae): does morphological specialization influence task plasticity?. Behav. Ecol. Sociobiol..

[2] Page RE, Amdam GV (2007). The making of a social insect: developmental architectures of social design. BioEssays.

[3] Seeley TD (1982). Adaptive significance of the age polyethism schedule in honeybee colonies. Behav. Ecol. Sociobiol..

[4] Pankiw T, Page RE, Kim Fondrk M (1998). Brood pheromone stimulates pollen foraging in honey bees (Apis mellifera). Behav. Ecol. Sociobiol..

[5] Pankiw T, Huang Z-, Winston M, Robinson G (1998). Queen mandibular gland pheromone influences worker honey bee (Apis mellifera L.) foraging ontogeny and juvenile hormone titers. J. Insect Physiol..

[6] Moore D (2001). Honey bee circadian clocks: behavioral control from individual workers to whole-colony rhythms. J. Insect Physiol..

[7] Ament SA, Corona M, Pollock HS, Robinson GE (2008). Insulin signaling is involved in the regulation of worker division of labor in honey bee colonies. Proc. Natl. Acad. Sci..

[8] Guidugli KR (2005). Vitellogenin regulates hormonal dynamics in the worker caste of a eusocial insect. FEBS Lett..

[9] Reim T, Scheiner R (2014). Division of labour in honey bees: age- and task-related changes in the expression of octopamine receptor genes. Insect Mol. Biol..

[10] Behrends A, Scheiner R, Baker N, Amdam GV (2007). Cognitive aging is linked to social role in honey bees (Apis mellifera). Exp. Gerontol..

[11] Seeley TD, Kolmes SA (2010). Age Polyethism for Hive Duties in Honey Bees - Illusion or Reality?. Ethology.

[12] Korst PJAM, Velthuis HHW (1982). The nature of trophallaxis in honeybees. Insectes Soc..

[13] Wainselboim AJ, Roces F, Farina WM (2002). Honeybees assess changes in nectar flow within a single foraging bout. Anim. Behav..

[14] Núñez, J. A. Quantitative Beziehungen zwischen den Eigenschaften von Futterquellen und dem Verhalten von Sammelbienen. *Z*. *Vgl*. *Physiol*. **53**, 142–164 (1966).

[15] Goyret J, Farina WM (2005). Trophallactic chains in honeybees: a quantitative approach of the nectar circulation amongst workers. Apidologie.

[16] Díaz PC, Grüter C, Farina WM (2007). Floral scents affect the distribution of hive bees around dancers. Behav. Ecol. Sociobiol..

[17] Gil, M. & Farina, W. M. Crop scents affect the occurrence of trophallaxis among forager honeybees. *J*. *Comp*. *Physiol*. A **189**, 379–382 (2003). 10.1007/s00359-003-0412-4 12720034

[18] Nieh JC (2010). A Negative Feedback Signal That Is Triggered by Peril Curbs Honey Bee Recruitment. Curr. Biol..

[19] Morse, R. A. & Nowogrodzki, R. *Honey bee pests*, *predators*, *and diseases*. *Honey bee pests*, *predators*, *and diseases*. (Cornell University Press 1990).

[20] Fry CH (1983). Honeybee Predation by Bee-Eaters, with Economic Considerations. Bee World.

[21] Ibanez S, Gallet C, Després L (2012). Plant Insecticidal Toxins in Ecological Networks. Toxins (Basel)..

[22] Dukas R (2001). Effects of perceived danger on flower choice by bees. Ecol. Lett..

[23] Lau CW, Nieh JC (2010). Honey bee stop-signal production: temporal distribution and effect of feeder crowding. Apidologie.

[24] Tedjakumala SR (2013). Rules and mechanisms of punishment learning in honey bees: the aversive conditioning of the sting extension response. J. Exp. Biol..

[25] Kessler D, Gase K, Baldwin IT (2008). Field experiments with transformed plants reveal the sense of floral scents. Science.

[26] Raguso RA (2004). Why are some floral nectars scented?. Ecology.

[27] Behrends A, Scheiner R, Baker N, Amdam G (2007). Cognitive aging is linked to social role in honey bees (Apis mellifera). Exp. Gerontol..

[28] Scheiner R, Amdam GV (2009). Impaired tactile learning is related to social role in honeybees. J. Exp. Biol..

[29] Huang MH, Seeley TD (2003). Multiple unloadings by nectar foragers in honey bees: a matter of information improvement or crop fullness?. Insectes Soc..

[30] Seeley TD (1989). Social foraging in honey bees: how nectar foragers assess their colony’s nutritional status. Behav. Ecol. Sociobiol..

[31] Heil M (2011). Nectar: generation, regulation and ecological functions. Trends Plant Sci..

[32] Kidawa D, Barcikowski M, Palme R (2017). Parent-offspring interactions in a long-lived seabird, the Little Auk (Alle alle): begging and provisioning under simulated stress. J. Ornithol..

[33] Tilgar V, Moks K, Saag P (2011). Predator-induced stress changes parental feeding behavior in pied flycatchers. Behav. Ecol..

[34] Caro SM, Griffin AS, Hinde CA, West SA (2016). Unpredictable environments lead to the evolution of parental neglect in birds. Nat. Commun..

[35] Galef BG, Stein M (1985). Demonstrator influence on observer diet preference: Analyses of critical social interactions and olfactory signals. Anim. Learn. Behav..

[36] Galef BG (1998). Familiarity and relatedness: Effects on social learning about foods by Norway rats and Mongolian gerbils. Anim. Learn. Behav..

[37] Valsecchi P, Choleris E, Moles A, Guo C, Mainardi M (1996). Kinship and familiarity as factors affecting social transfer of food preferences in adult Mongolian gerbils (Meriones unguiculatus). J. Comp. Psychol..

[38] McFadyen-Ketchum SA, Porter RH (1989). Transmission of food preferences in spiny mice (Acomys cahirinus) via nose-mouth interaction between mothers and weanlings. Behav. Ecol. Sociobiol..

[39] Czaczkes TJ, Grüter C, Ratnieks FLW (2013). Negative feedback in ants: crowding results in less trail pheromone deposition. J. R. Soc. Interface.

[40] Hrncir M (2006). Vibrating the food receivers: a direct way of signal transmission in stingless bees (Melipona seminigra). J. Comp. Physiol. A.

[41] Mc Cabe SI, Hrncir M, Farina WM (2015). Vibrating donor-partners during trophallaxis modulate associative learning ability of food receivers in the stingless bee Melipona quadrifasciata. Learn. Motiv..

[42] Wehmann H-N, Gustav D, Kirkerud NH, Galizia CG (2015). The Sound and the Fury—Bees Hiss when Expecting Danger. PLoS One.

[43] Arenas A, Farina WM (2008). Age and rearing environment interact in the retention of early olfactory memories in honeybees. J. Comp. Physiol. A.

[44] Provecho Y, Josens R (2009). Olfactory memory established during trophallaxis affects food search behaviour in ants. J. Exp. Biol..

[45] Smith, B. H. & Burden, C. M. A proboscis extension response protocol for investigating behavioral plasticity in insects: application to basic, biomedical, and agricultural research. *J*. *Vis*. *Exp*. **e51057**, 10.3791/51057 (2014). 10.3791/51057 PMC4828057 25225822

[46] Getz WM, Smith KB (1990). Odorant moiety and odor mixture perception in free-flying honey bees (*Apis mellifera*). Chem. Senses.

[47] Matsumoto Y, Menzel R, Sandoz J-C, Giurfa M (2012). Revisiting olfactory classical conditioning of the proboscis extension response in honey bees: A step toward standardized procedures. J. Neurosci. Methods.

[48] Page RE, Fondrk MK, Erber J (1998). The effect of genotype on response thresholds to sucrose and foraging behavior of honey bees (Apis mellifera L.). J. Comp. Physiol. A Sensory, Neural, Behav. Physiol..

[49] Team RDC (2016). & R Development Core Team, R. R: A language and environment for statistical computing. R Foundation for Statistical Computing, Vienna, Austria. R Foundation for Statistical Computing.

[50] GraphPad Prism Windows, GraphPad Software, La Jolla California USA.

[51] Jaeger TF (2008). Categorical Data Analysis: Away from ANOVAs (transformation or not) and towards Logit Mixed Models. J. Mem. Lang..

[52] Brooks, M. E. *et al*. *glmmTMB Balances Speed and Flexibility Among Packages for Zero-inflated Generalized Linear Mixed Modeling*.

